# Impact of the Influenza Vaccine on Myocardial Infarction Risk: A Systematic Review

**DOI:** 10.7759/cureus.97511

**Published:** 2025-11-22

**Authors:** Stephanie Nagy, Lily Tehrani, Aisha Abdool, David Israilov, Oksana Denis, Jeffrey Shin, Fayha Asim, Khadija Basharat, Kayvan Amini, Marc M Kesselman

**Affiliations:** 1 Rheumatology, Nova Southeastern University Dr. Kiran C. Patel College of Osteopathic Medicine, Davie, USA; 2 Rheumatology, Nova Southeastern University, Davie, USA; 3 Cardiology, Nova Southeastern University Dr. Kiran C. Patel College of Osteopathic Medicine, Davie, USA

**Keywords:** cardiac arrest, flu vaccine, heart attack, influenza, influenza type a, influenza type b, influenza type c, myocardial infraction, vaccination

## Abstract

Influenza continues to pose a significant global public health challenge. Older adults are particularly vulnerable to the virus, largely due to age-related declines in immune function and the presence of comorbidities such as hypertension and diabetes mellitus. Moreover, influenza can worsen existing health conditions. This systematic review examined the effect of influenza vaccination on the risk of developing myocardial infarctions. Influenza vaccine may play a role in reducing negative cardiovascular (CV) outcomes, especially if received* *annually. Due to the greater risk of influenza infection complications among older adults identified in this systematic review, especially CV disease complications, additional prospective studies evaluating risk mitigation with annual influenza vaccination are warranted, and the potential for awareness programs promoting annual influenza vaccination should be considered as a preventive health measure. This may help to significantly reduce both influenza-related illness and CV complications among high-risk populations, including elderly individuals.

## Introduction and background

Influenza, also referred to as the flu and caused by the influenza virus, remains a major public health concern globally. According to the World Health Organization, there have been an estimated 1 billion cases of seasonal influenza annually, with the 2024-2025 flu season having the highest numbers since 2009 [1]. During the 2023-2024 influenza season, the United States experienced approximately 40 million cases, with about 28,000 cases leading to death [2,3]. Certain demographic groups, including individuals over the age of 65 and children under the age of 2, exhibit heightened susceptibility to influenza infection [3]. The influenza virus can be categorized into influenza A, B, and C. Among these, A and B are subtypes that have been shown to cause severe pandemic and seasonal influenza, respectively. Influenza A has the ability to spread from migrating birds, pigs, horses, and humans, a significant factor in causing an epidemic and pandemic. In contrast to seasonal influenza, pandemics are responsible for increased morbidity and mortality. Influenza B causes only human-to-human spread with no other hosts involved and, therefore, is not involved in pandemics. Influenza C is a mild disease that commonly causes cold-like symptoms and sometimes causes lower respiratory infections that mainly spread between humans but have been occasionally detected in cattle and dogs [4-8].

Once entering the host and replicating, symptoms can begin to develop within 1-4 days. Signs and symptoms of mild cases of influenza include cough, fever, sore throat, myalgia, headache, runny nose, conjunctivitis, and a frontal or retro-orbital headache with photophobia [9]. The symptoms may progress into shortness of breath, tachycardia, hypotension, and the need for intubation or respiratory supportive care in more critical cases [9]. The interaction between the influenza virus and alveolar epithelial cells of the upper respiratory tract can drive the development of severe diseases such as acute respiratory distress syndrome and concomitant bacterial or viral pneumonia. Beyond these respiratory complications, the influenza virus is associated with extrapulmonary complications in multiple organs, such as cardiovascular (CV) (myocarditis, pericarditis, and tachycardia), neurological (encephalitis, encephalopathy, and meningitis), and ophthalmological complications (conjunctivitis, photophobia, and blurred vision), indicating its multi-organ pathology [10]. Meanwhile, with administration of the annual influenza vaccine prior to infection, the symptoms that the patients experience are far milder, and they are less likely to develop complications leading to hospitalization [11].

Some individuals are at an increased risk of perilous consequences that may compromise their immune systems and physical well-being. There are two main groups at risk for the complications of influenza: children (especially those under two years of age) and older adults. Children, notably infants, suffer from influenza complications since their immune systems have not yet fully matured [12]. Similar to children, older adults experience more severe impacts of the influenza virus due to their immune system progressively weakening, leading to complications such as pneumonia, or they are at higher risk due to their co-morbidities, such as hypertension and diabetes mellitus [13]. In recent years, older adults account for a significant portion of seasonal flu-related deaths (approximately 70%-80%) and represent 50%-70% of flu-related hospitalizations [14]. A study comparing hospitalization rates among children and adults aged 65 or older found that those who were elderly had a 9.4-fold increase in hospitalization [15]. It was also found that elderly adults were hospitalized on average three weeks earlier than children with complications of the infection [16]. This difference in hospitalization rates highlights an increasing concern about the severity of influenza among elderly adults, along with the narrowing time frame to effectively treat these patients.

Elderly adults, as a result of the influenza infection, can experience exacerbations of comorbid conditions and/or the development of novel complications following infection. A major concern following influenza infection within this population is the impact it may have on the CV system. Cardiovascular disease (CVD), especially cardiac ischemia, congestive heart failure, and cerebrovascular accidents, serves as the primary cause of mortality among adults aged 65 and older [17]. The ageing process triggers a cascade of physiological changes that increase an individual’s susceptibility to CVD. Progressive arterial stiffness, driven by elastin degradation and collagen deposition, can be associated with elevated systolic blood pressure and promotes left ventricular hypertrophy [18]. Atherosclerosis, a primary cause of coronary artery disease (CAD), worsens with age since increased endothelial dysfunction and oxidative stress limit vasodilation, raising the risk of transient ischemic episodes [19]. Ultimately, the accumulation induces inflammatory signals by binding to receptors such as the receptor for advanced glycation end products to initiate an activation pathway toward CVD complications [20]. This promotes the development of "inflammaging," a chronic and mild inflammation responsible for limiting atherosclerotic plaque stability and stimulating thrombosis [21]. The accumulation of DNA damage, which causes genomic instability, also reduces the function of the endothelium and promotes the development of atherosclerosis [22]. At the same time, these age-related changes considerably increase the risk for these CV conditions in older individuals.

It has been found that even mild influenza infections can be associated with a doubled risk of acute CV events among older patients [23]. Pro-inflammatory cytokines play a myriad of roles related to CV complications. Tumour necrosis factor-alpha (TNF-α) and interleukin-1 beta (IL-1β) have been shown to play integral parts in systemic inflammation and endothelial dysfunction, while interleukin-6 (IL-6) and interleukin-8 (IL-8) have been demonstrated to contribute to vascular resistance and thrombosis [24]. During an influenza infection, pro-inflammatory cytokines of TNF-α, IL-1β, IL-6, and IL-8 have been shown to be released, which disrupt endothelial function, raise vascular resistance, and increase the risk of thrombosis, which subsequently increases the likelihood of experiencing CV complications such as a myocardial infarction (MI), stroke, or heart failure [25]. This increased inflammatory response, coupled with influenza infection’s pro-thrombotic consequences, has been seen to exacerbate CV complications among the elderly, often resulting in hospitalisation or death [26].

The influenza vaccine may reduce MI risk by limiting systemic inflammation and its effects on atherosclerosis. Influenza infection triggers a surge in cytokines like IL-1β, IL-6, and TNFα, which contribute to endothelial dysfunction, increased monocyte recruitment, and macrophage infiltration into plaques, making them more prone to rupture. The infection also elevates matrix metalloproteinases (MMP-9 and MMP-13), which can degrade the fibrous cap of plaques, further increasing instability. By preventing infection, the vaccine reduces these inflammatory and vascular changes, lowering the likelihood of plaque rupture and thrombosis, key events in MI. In addition, vaccination has been associated with reduced levels of IFNγ, IL-2, and TNFα, suggesting a broader role in modulating inflammation linked to CV risk.

Research efforts have demonstrated that the most effective way to reduce the development of influenza and prevent complications is through vaccination. The Advisory Committee on Immunization Practices and the Centers for Disease Control recommend routine annual influenza vaccination for all individuals aged six months and older who do not have contraindications [27]. For the 2023-2024 influenza season, vaccine effectiveness was found to be 59%-67% among children and 33%-49% among adults [28].

Older individuals are at an increased risk of complications associated with their influenza infection, in addition to their elevated CV complications related to aging. Vaccinated patients indicate a lower risk of all-cause mortality and CV mortality [29]. As such, this systematic review aims to better understand the effectiveness of the annual influenza vaccine in reducing the rates of MI among older adults, especially the elderly. 

## Review

Search strategy

A systematic literature review was performed on March 18th, 2025, using Ovid (MEDLINE), CINAHL, PUBMED, and Web of Science using the search terms (“Influenza OR Influenza Virus Type A OR Influenza Virus Type B OR Influenza Virus Type C OR Influenza Vaccine OR Flu vaccine”) AND (“Myocardial infarction OR heart attack OR STEMI OR ST Elevated Myocardial Infarction OR NSTEMI OR Non ST Elevated Myocardial Infarction OR myocardial ischemia.”) To ensure the recency of the articles, only articles published between 2010 and 2025 were assessed. To ensure the quality of articles, only peer-reviewed articles were included in the searches. Duplicates were identified and removed via the Rayyan screening system. The articles were analyzed in a stepwise process by first evaluating the title and abstract for relevance and then assessing the full-text manuscript. The Nova Southeastern University library database was utilized to access databases and full-text articles.

Selection criteria

**Figure 1:**
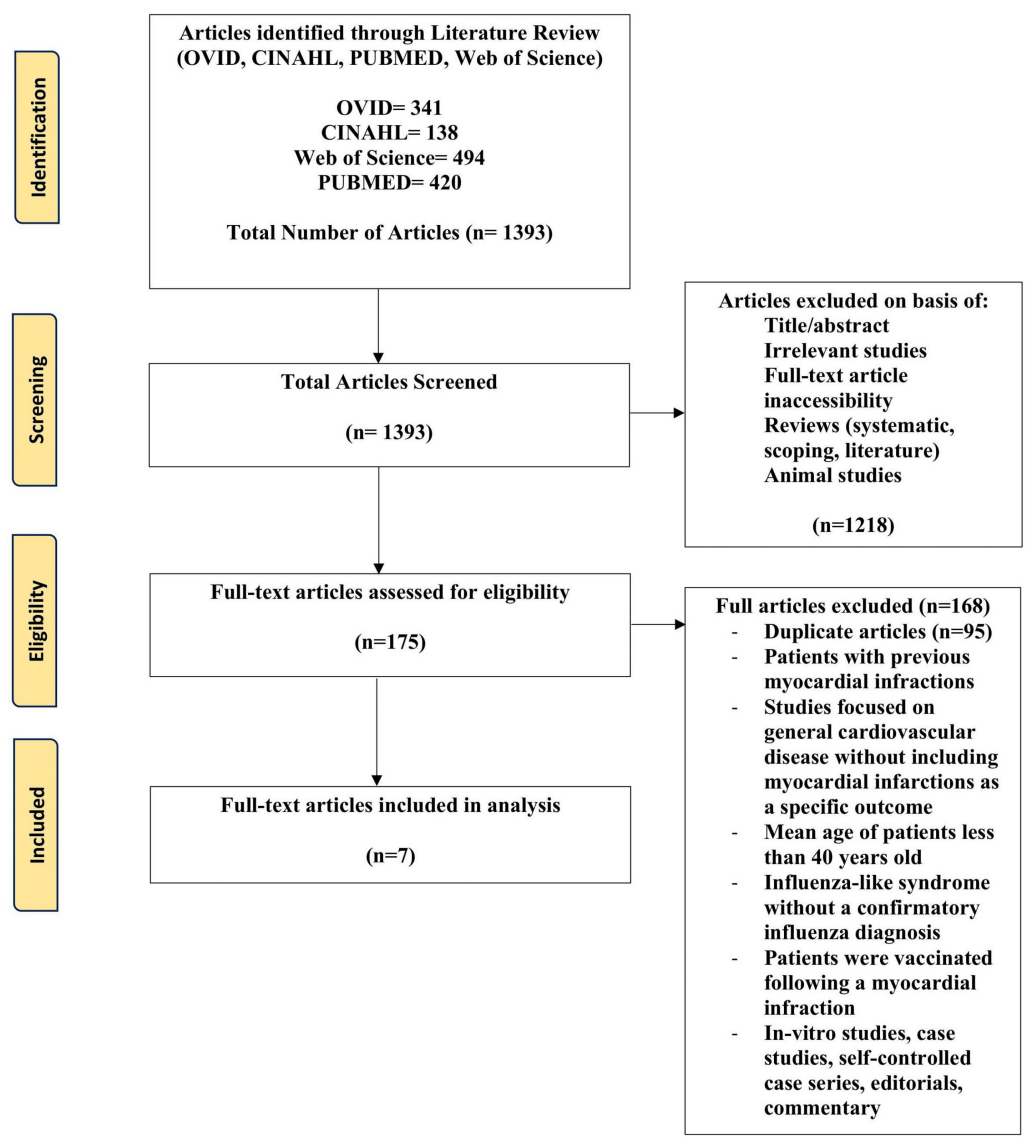
Preferred reporting items for systematic reviews and meta-analyses flow diagram for article selection PubMed: Public Medical Database, CINAHL: Cumulative Index to Nursing and Allied Health Literature

For this review, we included randomized controlled trials, cross-sectional studies, observational studies, and cohort prospective/retrospective studies. The population included patients above 40 years of age without prior MI who received the influenza vaccine. The primary outcome measure was whether annual influenza vaccination prevented first-time MIs. Studies excluded from this review were literature, systematic, or scoping reviews; animal studies; case studies; editorials; commentary; ecological studies; self-controlled case series; and in-vitro-focused outcomes. Articles were excluded if the patients had previous MIs, if the influenza vaccine was given post a CV event in observation of sequelae conditions, if studies failed to specify MIs as an outcome, if patients had influenza-like syndrome but were not diagnosed with influenza, or if the patients were under 40 years of age to ensure a focus on the adult and elderly population. Articles not written in English were excluded, as English is the primary language of the authors. Hand searching was not utilized to add articles to the analysis. Two reviewers completed a blinded review process of the articles to decide on their inclusion or exclusion based on the determined criteria, and a third reviewer was used to break any ties. No third reviewer was required to tie-break. The preferred reporting items for systematic reviews and meta-analyses (PRISMA) guidelines were followed and used to develop a flow diagram of the selection criteria for reproducibility (Figure 1) [30]. Preferred reporting items for systematic reviews and meta-analyses flow diagram for article selection.

Critical appraisal

The included studies were assessed for risk of bias using the Cochrane Risk of Bias in Non-randomized Studies of Interventions (ROBINS-I) tool (Table 1).

**Table 1:** Risk of bias assessment using the Cochrane ROBINS-I tool Risk of bias for each included study was assessed using the Cochrane risk of bias in non-randomized Studies of Interventions (ROBINS-I) tool. Seven domains were evaluated: bias due to confounding, bias in classification of interventions, bias in selection of participants, bias due to deviations from intended interventions, bias due to missing data, bias in measurement of outcomes, and bias in selection of reported results. Each domain was rated as low, moderate, serious, or critical risk of bias, and an overall rating was determined based on the highest level of concern identified across domains.

Study (Author, Year)	Bias due to Confounding	Bias in Classification of Interventions	Bias in Selection Participation	Biased due to Deviations from Intended Interventions	Bias due to Missing Data	Bias in Measurement of Outcomes	Bias in Selection of Reported Results	Overall Risk of Bias
Streeter et al. (2022)	Low	Low	Low	Moderate	Low	Low	Low	Low-Moderate
De Abajo et al. (2022)	Low	Low	Low	Moderate	Low	Low	Low	Low-Moderate
MacIntyre et al. (2013)	Moderate	Moderate	Moderate	Moderate	Moderate	Low	Moderate	Moderate
Chiang et al. (2017)	Low	Low	Low	Moderate	Low	Low	Low	Low
Modin et al. (2022)	Low	Low	Low	Moderate	Low	Low	Low	Low
Siriwardena et al. (2010)	Low	Moderate	Low	Moderate	Low	Low	Low	Low-Moderate
Lavallée et al. (2014)	Moderate	Moderate	Moderate	Moderate	Moderate	Low	Moderate	Moderate

Results

In total, 1,393 articles were populated, with seven meeting the inclusion criteria for this systematic review. Table 2 depicts the findings of the included studies, including the number and sex of patients, mean age, comorbidities, timing of when the vaccine was received and number of vaccinations, follow-up time monitoring for MI, and the overall findings on MI risk.

**Table 2:** Findings of the studies analyzed in the review HTN: Hypertension, CHD: Coronary heart disease, MI: Myocardial infraction, CVA: Cerebral vascular event, OA: Obstructive sleep apnea, COPD: Chronic obstructive pulmonary disease, CKD: Chronic kidney disease, MACE: Major adverse CV event, CAD: Coronary artery disease, HIV: Human immunodeficiency virus, OR: Odds ratio, CI: Confidence interval, M: Male, F: Female

Author	Number and sex of patients (M= male, F= female)	Mean age (years)	Comorbidities	Vaccine Timing / Follow-up	Key Findings	Main Conclusion
Streeter et al. [31]	62,644 to 130,460 annual cohorts; 41.9–45.2% male	Decreased 1997–2011 from 74.3 to 69.9 years old	HTN (36.43%), CHD (12.63%), depression (8.21%), asthma (6.79%), hypothyroidism (6.19%), stroke (5.77%), cancer (5.64%), diabetes mellitus (5.27%), atrial fibrillation (5.26%), COPD (4.83%), CKD (4.70%), mental health conditions, dementia, or epilepsy (4.29%), and heart failure (3.67%)	Annual vaccination Sept 1 – Jan 31; 12-mo follow-up; flu seasons 1997 – 2011 (UK)	Influenza vaccination reduced MI risk by 39% overall (95% CI 34–44%); effectiveness against MI often greater than against influenza itself (esp. 2003, 2007, 2010–11 seasons)	Annual influenza vaccination offers moderate protection against MI in adults ≥ 65 years old; effect varies by season but remains protective.
De Abajo et al. [32]	24,155 cases (17,208 M, 6947 F) + 120,775 controls (86,040 M, 34,735 F)	67.1 (cases); 67.0 (controls)	Smoking (n=6,498), ischemic CVA (n=600), heart failure (n=909), angina pectoris (n=2,735), peripheral artery disease (n=1,092), hypertension (n=12,534), diabetes mellitus (n=6,543), dyslipidemia (n=11,355), COPD (n=1,989), RA (n=238), OA (n=2,157), CKD (n=919), gout (n=1,164)	≥ 14 days before MI index date with same season; Sept 1 – Aug 31 each year; seasons 2002 – 2015 (Spain)	Vaccination associated with 10–15% reduced MI risk (AOR 0.85, 95% CI 0.82–0.88); protective effect consistent across epidemic, pre-, and post-epidemic periods	Influenza vaccination serves as primary prevention for CV events, including MI, across age and risk groups.
MacIntyre et al. [33]	275 cases (216 M, 59 F); 284 controls (130 M, 154 F)	64% of cases aged 40-64 years old, 36% of cases aged ≥ 65 years old, 25.4% of controls aged 40-64 years old, 74.6% of controls aged ≥ 65 years old	Hypercholesteremia (n=150), hypertension (n=148), diabetes (n=68), COPD (n=41)	Three influenza seasons 2008, 2009, 2010; 4 – 6 week follow-up (Austrailia)	Influenza vaccination protective against MI (OR 0.55, 95% CI 0.35–0.85); vaccine effectiveness 45% (95% CI 15–65%) ages 40–64 years old, 33% (–20 to 63%) ≥ 65 years old	Recent influenza infection was common but not directly predictive of MI after adjustment. Influenza vaccination significantly reduced the risk of MI
Chiang et al. [34]	80,363 (44,737 M, 35,626 F)	76.8	Hypertension (n=68,096), CAD (n=42,909), diabetes mellitus (n=38,383), dyslipidemia (n=37,349) heart failure (n=19,196), CKD (n=18,920), cancer (n=12,435), valvular heart disease (n=11,421), atrial fibrillation (n=7022), and peripheral vascular disease (n=6,157)	Vaccination in prior year before index date; 4-y follow-up; seasons 2000 – 2013 (Taiwan)	Vaccination had significant protective effect against MI (OR 0.80, 95% CI 0.76–0.84, p < 0.001)	Influenza vaccination in the previous year associated with reduced risk of primary MACE including MIs.
Modin et al. [35]	608,452 (266,683 M, 341, 769 F)	65.2	Valvular disease (n=10,132), systemic embolus (n=3,160), atrial fibrillation or flutter (n=33,291), chronic renal failure (n=8080), anemia (n=10,477), diabetes (n=79,822) peripheral vascular disease (n=9199), liver disease (n=5703), rheumatic disease (n=9,739), and peptic ulcer (n=13,619)	Vaccinated Sept–Nov before each flu season (Dec 1 – Apr 1); median follow-up 5 seasons; 2007 – 2016 (Denmark)	Risk of MI or stroke-related death reduced in vaccinated patients (p = 0.017); decreased all-cause death (95% CI 0.81–0.86, p < 0.001); decreased CV death (0.82–0.88, p < 0.001); decreased stroke/MI death (0.88–0.99, p = 0.042)	Influenza vaccination significantly reduces all-cause and CV mortality in patients with hypertension.
Siriwardena et al. [36]	78,706 patients 16,012 cases (6168M, 9844 F) 62,694 controls (24171 M, 38523 F)	66.6 (cases); 65.9 (controls)	Asthma or COPD attack (n=1,980), chronic heart disease (n=3,756), stroke or transient ischemic attack (n= 1,434), diabetes (n=2,451), splenectomy (n=36), chronic liver disease (n=30), chronic renal disease (n=584), immunosuppression or HIV (n=2)	Vaccination in previous year; early (Sept–mid-Nov) vs. late (mid-Nov–Feb); Nov 2001 – May 2007 (UK)	19% decreased first-time MI in those above 40 (adjusted OR 0.81, 95% CI 0.77–0.85, p < 0.001); early vaccination had a 21% decreased vs. 12% decreased in late vaccinations (p = 0.042)	Influenza vaccination administered early in the season, was associated with a lower risk of first-time acute myocardial infarction
Lavallée et al. [37]	23,110 (13,935 M, 9,175 F)	70 (vaccinated) vs. 65.5 (non-vaccinated)	Hypertension (n=4,179), diabetes mellitus (n=1,492), dyslipidemia (n=2,600), current smoking (n=974), known CV disease (n=1,075), known coronary artery disease (n=911), known peripheral artery disease (n=221)	Follow-up 1, 3, 6 mo and every 6 mo thereafter; ≥ 2 y; seasons 2004 – 2010 (France)	Reduction of MI risk 95% CI 0.59–1.18 (p = 0.30); no significant association for stroke or composite vascular events	Influenza vaccination did not significantly reduce the risk of major vascular events

Following vaccination, the follow-up time monitoring for MI events varied from 4-6 weeks up to six years. All studies concluded that influenza vaccines significantly reduced the risk of acute MIs, except Lavallee et al., who noted a reduction in risk, but the finding was not significant. Siriwardena et al. was the only study to compare the impact of receiving influenza early or late into the flu season. It was concluded that early vaccinations showed greater reductions in MI risk as compared with vaccinations later in the season.

It is important to note that, due to CV comorbidities increasing with age, the complete absence of patients without any form of CV diagnosis was not possible. Meanwhile, all studies that included patients with previous MIs were excluded. Comorbidities that were the most prevalent among patients across studies included hypertension, diabetes mellitus, dyslipidemia, and chronic heart disease.

Only three studies contained a breakdown of the impact of influenza vaccination on patients with established comorbidities. Specifically, De Abajo et al. concluded that vaccinations reduced MI by 10%-15%. They further found that this finding remained consistent among patients with low, intermediate, and high CV risk factors (CI: 0.84, 0.90, and 0.86, respectively). Chiang et al. noted that vaccinations had protective effects for patients against MIS. In addition, the researchers found that major adverse CV events (MACE) within the whole study population were reduced (CI 0.78 (0.77-0.80), p<0.001). Modin et al. found an MI reduction following vaccination (p = 0.017). In addition, the investigators identified an association between influenza vaccination and a reduced risk for all-cause mortality among patients with hypertension (HR, 0.82; 95% CI, 0.79-0.85; p<0.001).

An in-depth analysis was conducted on each study, as outlined below:

Streeter et al. conducted a quasi-experimental study design over a 15-year period (1997-2011) among UK adults aged ≥ 65 years old to estimate the effectiveness of the influenza vaccine in reducing the risk of MI. Annual cohorts had no record of influenza vaccination in the prior influenza season and were considered vaccinated if they had a recorded vaccination between September 1 and January 31 of each flu season. Patient follow-up was completed 14 days after vaccination and followed for up to one year or until death, vaccination, or study withdrawal. The prior event rate ratios (PERR) method was utilized in order to control for confounding variables. Influenza vaccination was associated with a 39% decrease in MI risk (PERR-adjusted HR: 0.61; 95% CI: 0.56-0.66). Patients who received the vaccination also showed variable yet protective effects against the influenza virus, with PERR-adjusted HR ranging from 0.70 in 1999 (30% effectiveness) to 0.99 in 2001 (1% effectiveness). In comparison, vaccine effectiveness against MIs was more pronounced with PERR-adjusted HR ranging from 0.40 in 2010 (60% effectiveness) to 0.89 in 2001 (11% effectiveness). Although researchers found an overall reduction in MI risk, certain years showed higher protection against MIs in comparison to influenza, which suggests non-influenza-mediated mechanisms affecting risk. In 2003, 2007, and 2010-2011, influenza vaccination was found to be more effective in the prevention of MIs as compared with influenza, suggesting non-influenza-mediated CV protection [31].

In a population-based case-control study using the Spanish BIFAP database, De Abajo et al. investigated the association between influenza vaccination and the risk of a first acute MI between 2002 and 2015. The researchers included 24,155 validated first-time MI patients and 120,775 age-, sex-, and index date-matched controls. Patients ranged from 40 to 99 years of age. The investigators found that the crude odds ratio (OR) for influenza vaccination and MI was 1.01 (95% CI: 0.98-1.05), suggesting no protective effect prior to adjustment. After adjusting for comorbidities and medications, the adjusted odds ratio (AOR) decreased to 0.85 (95% CI: 0.82-0.88), indicating a 15% decrease in the risk of a first MI following influenza vaccination. Risk reduction was found to be similar among all 3 epidemic periods assessed (pre-epidemic AOR = 0.87, 95% CI: 0.79-0.95; epidemic AOR = 0.89, 95% CI: 0.82-0.96; and post-epidemic AOR = 0.83, 95% CI: 0.79-0.87) and appeared soon after vaccination (15-30 days) and remained stable over time. Protective effects were also found to be similar across subgroups, including sex, age group (< 65 vs. ≥ 65), and CV risk stratification (low, intermediate, and high), with no statistically significant interaction observed between vaccination and subgroup variables. In addition, vaccinated patients were found to have higher CV comorbidities and medication use compared to the unvaccinated patients. Although vaccinations primarily took place between weeks 38 and 49, which coincided with the seasonal flu, many of the protective effects were seen outside of the main influenza waves, pointing toward mechanisms unrelated to direct influenza prevention [32].

In a prospective case-control study, MacIntyre et al. evaluated whether influenza vaccinations were protective against acute MIs. Participants were followed over 3 consecutive influenza seasons (2008-2010). A total of 559 participants aged ≥ 40 years were included in the study, with 275 acute MI cases and 284 healthy controls. A total of 9.5% of participants had laboratory-confirmed influenza infection, with MI cases having a significantly higher proportion (12.4%) as compared with healthy controls (6.7%) (OR: 1.97, 95% CI: 1.09-3.54, p= 0.022). Meanwhile, after controlling for confounding variables, influenza infection was no longer a significant predictor of acute MI (adjusted OR: 1.07, 95% CI: 0.53-2.19, p = 0.849). Vaccination against influenza during the recruitment was significantly protective (AOR: 0.55, 95% CI: 0.35-0.85, p = 0.008), which translates to a vaccine effectiveness (VE) against acute MI of 45% (95% CI: 15%-65%). The estimated VE against MI was 33% (95% CI: -20%-63%) for participants aged ≥ 65 and 45% (95% CI: -15%-73%) for individuals aged 40 to 64. The efficacy of the vaccine against influenza infection itself was 83.6% (95% CI: 27.6%-96.3%). The researchers also found that there may have been an underdiagnosis of influenza infection in patients with acute MI, as many laboratory-confirmed influenza cases were not clinically recognized at the time of hospital admission [33].

In a large-scale population-based study, conducted between 2000 and 2013, Chiang et al. expanded the scope of their research to include MACEs. The researchers included 160,726 elderly adults (≥ 65 years) to assess the potential protective effects of influenza vaccination. Cases were defined as patients who had a new diagnosis of MACE over the study period, which included acute MIs and ischemic strokes. The investigators analyzed 80,363 MACE cases, along with 80,363 age- and sex-matched controls. Cases were significantly more likely to have used several medications, such as non-steroidal anti-inflammatory drugs (NSAIDs), calcium channel blockers (CCBs), and insulin (p < 0.001). Researchers found that influenza vaccination significantly reduced the odds of overall MACEs (AOR: 0.80, 95% CI: 0.78-0.82, p<0.001), MI (AOR: 0.80, 95% CI: 0.76-0.84, p<0.001), and ischemic strokes (AOR: 0.80, 95% CI: 0.77-0.82, p<0.001), translating to a 20% reduction in risk. In addition, influenza illness was associated with an increased risk of overall MACEs (AOR: 1.24, 95% CI: 1.18-1.29, p<0.001), MI (AOR: 1.46, 95% CI: 1.34-1.59, p<0.001), and ischemic stroke (AOR: 1.16, 95% CI: 1.10-1.22, p<0.001). Among patients that experienced infection, those who were vaccinated showed no significant increased risk of MACES (AOR: 0.99, 95% CI: 0.92-1.07, p=0.834), MI (AOR: 1.05, 95% CI: 0.92-1.21, p=0.440), and/or ischemic stroke (AOR: 0.96, 95% CI: 0.89-1.05, p=0.398). This protective effect was found to be consistent among all subgroups, including sex (AOR: 0.78-0.82), age, and comorbidity stratification. Furthermore, patients who received the influenza vaccination in the prior year were found to have a more protective effect against ischemic stroke (AOR: 0.78, 95% CI: 0.75-0.81) as compared to those who did not receive prior vaccination (AOR: 0.82, 95% CI: 0.79-0.85) [34].

In a nationwide cohort study conducted by Modin et al., 608,452 patients with hypertension were followed for nine influenza seasons (2007 to 2016) to investigate the effect of influenza vaccination on mortality. After adjusting for confounders, influenza vaccination was associated with a significant reduction in all-cause mortality (HR: 0.82, 95% CI: 0.79-0.85, p<0.001), CV mortality (HR: 0.84, 95% CI: 0.80-0.89, p<0.001), and death from acute MI or stroke (HR: 0.90, 95% CI: 0.82-0.98, p=0.017), and the number needed to treat (NNT) to prevent 1 death over 1 season was 977 (95% CI: 837-1172). In addition, this protective effect was significantly stronger in preventing all-cause death (HR: 0.81, 95% CI: 0.78-0.85) and CV death (HR: 0.84, 95% CI: 0.80-0.89) in patients ≥ 65 years, with no significant effect found among patients < 65 years of age. This protective effect was found to be the greatest between December and March and lasted into the off-season [35].

In line with prior studies demonstrating the CV advantages of influenza and pneumococcal vaccination, Siriwardena et al. examined its correlation with lowering first-time MIs in a UK-based case-control study. The researchers analyzed 78,706 participants (16,012 acute MI cases and 62,694 matched controls) aged ≥ 40 years between 2001 and 2007. Researchers found that influenza vaccination within the past year was correlated with a 19% reduction in the rate of first-time acute MI (AOR: 0.81, 95% CI: 0.77-0.85, p<0.001). A repeated analysis using multiple imputation to account for missing values in body mass index (BMI), cholesterol, and blood pressure (BP) had similar results (AOR: 0.83, 95% CI: 0.80-0.88). This protective effect was seen in patients <65 years (OR: 0.81, 95% CI: 0.73-0.90) and ≥ 65 years (OR: 0.79, 95% CI: 0.75-0.83). The timing of vaccination also affected protective effects, as early season vaccination (September to November) had stronger protection (AOR: 0.79, 95% CI: 0.75-0.83) compared to late season vaccination (mid-November to February; AOR: 0.88, 95% CI: 0.79-0.97). Early vaccination continued to be substantially more protective than late vaccination (AOR: 0.90, 95% CI: 0.82-1.00, p = 0.042). The amount of time since the last vaccination also affected the protective effect, with smaller intervals of time correlating with greater protective benefits. Times ranged from 0-3 months (AOR: 0.80, 95% CI: 0.74-0.86), 3-6 months (AOR: 0.82, 95% CI: 0.76-0.89), and 6-12 months (AOR: 0.87, 95% CI: 0.81-0.94). After 12 months, no protective effects were observed (OR ≥ 1). In addition, the protective effects also differed between calendar months, with stronger associations between September and November (AOR: 0.75, 95% CI: 0.68-0.83), followed by April to August (AOR: 0.80, 95% CI: 0.73-0.86), and December to March (AOR: 0.86, 95% CI: 0.79-0.93). Participants who received repeated vaccinations over 5 to 6 prior seasons were found to have an even greater protective benefit (AOR: 0.79, 95% CI: 0.74-0.84). Furthermore, vaccination target groups had much stronger protective effects (AOR: 0.70, 95% CI: 0.64-0.77) than non-target groups (AOR: 0.85, 95% CI: 0.79-0.91, p < 0.001) [36].

Lavallée et al. assessed the impact of influenza vaccination on CV outcomes in patients with recent ischemic stroke or transient ischemic attacks (TIA). A pooled analysis was conducted from three large prospective studies, including 23,110 patients. The primary composite outcome of vascular death, MI, or stroke did not significantly differ between vaccinated (10.1%) and unvaccinated (10.0%) patients after propensity score matching (n=5,054; HR: 0.97, 95% CI: 0.85-1.11, p = 0.67). Similarly, no significant correlations were found between vaccination and individual outcomes of stroke (HR: 1.01, 95% CI: 0.88-1.17, p = 0.89) or MI (HR: 0.84, 95% CI: 0.59-1.18, p = 0.30). Given the sample size of 23,110 patients, the lack of statistical significance is unlikely to be due to sample size and more likely to be a result of study design or event rate. The primary outcome was nonfatal MI, nonfatal strokes, and vascular death up to two years of initial presentation. The primary outcomes exclude just under eighty per cent of patients initially enrolled. Propensity score-adjusted analysis of the full cohort also revealed no significant associations for the primary composite outcome (HR: 1.00, 95% CI: 0.89-1.12, p=0.99), stroke (HR: 1.03, 95% CI: 0.91-1.16, p=0.66), or MI (HR: 0.82, 95% CI: 0.62-1.08, p=0.15). In addition, no significant differences were found in subgroup analyses between age, sex, stroke, TIA, and history of CAD [37].

Discussion

The influenza vaccine has been shown to be an effective preventive measure for older adults, especially those who face a higher risk of severe illness from the virus due to the natural weakening of the immune system with age and the presence of comorbidities like diabetes and hypertension, which can exacerbate the infection's severity [13]. In recent years, this population accounts for a significant proportion of seasonal flu-related deaths, ranging from 70% to 80%, and represents 50% to 70% of flu-related hospitalizations [14]. One major concern with influenza infection in the elderly is its potential impact on the CV system. CV disease remains the leading cause of mortality among individuals aged 65 years and older, with ischemic heart disease, congestive heart failure, and cerebrovascular accidents as key underlying causes [17]. Influenza vaccination has been shown to help mitigate systemic inflammation and vascular stress caused by the virus, potentially reducing the risk of CV complications in this vulnerable group.

All studies reviewed indicated a reduced MI risk and demonstrated the potential additional benefit of the influenza vaccine besides its initial purpose to reduce the severity of influenza infection. As a result, a greater emphasis should be placed on ensuring timely annual influenza vaccination of the elderly population. Upon these findings, this warrants further investigation to confirm the CV and other additional benefits for elderly adults and encourage annual vaccination among this population. The Centers for Disease Control and Prevention (CDC) and the Advisory Committee on Immunization Practices recommend the use of higher-dose flu vaccines, such as high-dose inactivated and recombinant or adjuvanted inactivated vaccines, for individuals aged 65 years and above [14]. In the U.S., the recommended time for vaccination is September or October, with the ideal goal for all individuals to receive their vaccination by the end of October [14]. Nearly all studies, except for one, found a reduction in MI occurrence among older adults after receiving the influenza vaccine [31-37]. Specifically, Streeter et al. even noted that the influenza vaccine had greater effectiveness against reducing MI than acute influenza infections. Siriwardena et al. was the only study to analyze the timing of vaccinations. The researchers found that early-season vaccinations had greater protectiveness as compared with late-season vaccinations (p=0.042) [36].

Analyzing the usefulness of annual influenza vaccines on MI risk is not only crucial to limit CV risk due to the impact that the influenza virus can have on the CV system, but also to prevent the negative impacts on physical and mental health and quality of life (QOL). After a first MI, patients are at increased risk for CV complications, such as recurrent MI, congestive heart failure (CHF), arrhythmias, angina, stroke, and even death. In addition, complications during hospitalization, such as cardiogenic shock, atrial fibrillation, heart failure, stroke, and non-CV issues, were found to be proportional to age and increased with hospitalization [38]. Furthermore, the 30-day and one-year mortality rates were significantly higher in the older elderly patients (those 85 and older) as compared with their younger counterparts (those 65-69 years old) [38]. Following an acute MI, elderly patients often experience mobility impairments and a decline in activities of daily living, such as bathing, dressing, and walking around the home [39]. MI survivors reported lower health-related QOL in areas such as general health, physical health, daily activities, and mental health compared to matched healthy controls without a history of MI [40]. This leads to further comorbidity issues. These challenges can result in increased dependence on caregivers and reduced autonomy. Receiving the influenza vaccine may not only reduce infection, but it may also have downstream benefits by reducing the complications following an MI.

Even with the beneficial impact of the influenza vaccine, the influenza vaccination coverage remains suboptimal, particularly among those under 65 years of age. In the United States, for the 2023-2024 season, influenza vaccine uptake was 46.2% among individuals aged 50-64 and 69.7% among those aged 65 and older [41]. Influenza vaccine uptake may be improved through 4 suggested strategies: patient-centered, provider-centered, practice-centered, and policy-centered interventions [42]. Patient-focused approaches emphasize education, reminders, and financial incentives such as reducing or eliminating out-of-pocket costs. Provider-centered strategies involve training, reminders, and vaccination audits. Practice-based efforts include identifying eligible patients and extending vaccination campaigns. Policy interventions focus on incentivizing vaccine production, increasing demand through education, and implementing mandates. In addition, offering vaccinations in non-traditional settings, such as mobile clinics, can help improve accessibility, especially for underserved populations [42].

Addressing vaccine hesitancy is crucial. The Strategic Advisory Group of Experts on Immunization (SAGE) defines vaccine hesitancy as the delay or refusal of vaccination, despite the availability of vaccination services. It is a complex and context-dependent issue, influenced by various factors, including complacency, convenience, and confidence [43]. "Complacency" refers to a low perceived risk of infection and the belief that the disease is not a significant threat. Convenience encompasses barriers to healthcare access and affordability, while confidence reflects a lack of trust in authorities and skepticism about vaccine efficacy. More recently, a 4th factor, calculation, has been added, which involves assessing the perceived risk of disease and weighing the individual and society benefits of vaccination [42]. These 4 factors, complacency, convenience, confidence, and calculation, form the 4C model of vaccine hesitancy [42]. This model highlights the multifaceted nature of vaccine hesitancy, demonstrating that no single reason drives an individual’s reluctance toward vaccination. Instead, a combination of social, economic, political, and personal beliefs shapes vaccine decisions. To improve vaccination rates, it is essential to enhance vaccine accessibility and raise awareness of the risks associated with influenza infection, especially its potential to trigger heart attacks and other CV issues. Targeted public health campaigns, increased access to vaccines, and addressing vaccine hesitancy are crucial steps in preventing influenza-related CV events and improving overall public health.

The limitations of this research mainly revolve around the comorbidity status of patients. The population of patients who most commonly experience ischemic cardiac events is also likely to have other comorbid conditions that significantly impact their health. As seen within the articles included in this review, a large portion of patients had one or more CV-related comorbidities, making it difficult to completely conclude influenza vaccine efficacy in reducing MI, as the patients had co-existing comorbidities. Establishing a study that includes healthy patients with no CV comorbidities is a recommended next step to better understand the true protective effects of the influenza vaccine on this system. Additionally, many of the studies included were cohort studies. As randomized controlled trials are a gold standard, it is recommended to conduct additional studies where healthy age-matched adult and elderly patients receive or do not receive the influenza vaccine to assess its efficacy in reducing CV complications. It would be interesting to further examine a subpopulation with CAD who has no prior event to determine if receiving the vaccination helped to limit future cardiovascular events. Furthermore, the studies did not state which form of the influenza vaccine the patients were given; providing this information could allow for further investigation if one form of the vaccine is more effective. Future meta-analyses are recommended to be completed to assess the statistical significance among the studies.

A secondary hypothesis we would like to propose for future research studies is investigating how the low efficacy of the influenza vaccine has impacted atherothrombotic events. The efficacy of the influenza vaccine has been estimated to be between 19% and 60% from the 2009 to the 2024 flu seasons [44]. Potentially, if the efficacy of the influenza vaccine were elevated, would the rates of CV events decline even further in this population?

## Conclusions

Given the strong correlation between influenza infection and acute CV events, additional studies are warranted to help provide support for promoting annual flu vaccination. There is a potential that the use of influenza vaccines may reduce the occurrence of MIs, particularly among high-risk populations such as the elderly. By increasing awareness and uptake of the influenza vaccine through targeted health promotion efforts, especially in older adults, healthcare providers can play a crucial role in not only preventing seasonal flu but also helping to reduce the burden of CV complications.
